# Adipose Tissue-Derived Mesenchymal Stromal Cells Protect Mice Infected with *Trypanosoma cruzi* from Cardiac Damage through Modulation of Anti-parasite Immunity

**DOI:** 10.1371/journal.pntd.0003945

**Published:** 2015-08-06

**Authors:** Debora B. Mello, Isalira P. Ramos, Fernanda C. P. Mesquita, Guilherme V. Brasil, Nazareth N. Rocha, Christina M. Takiya, Ana Paula C. A. Lima, Antonio C. Campos de Carvalho, Regina S. Goldenberg, Adriana B. Carvalho

**Affiliations:** 1 Instituto de Biofísica Carlos Chagas Filho, Universidade Federal do Rio de Janeiro, Rio de Janeiro, Rio de Janeiro, Brazil; 2 Departamento de Radiologia, Hospital Universitário Clementino Fraga Filho, Rio de Janeiro, Rio de Janeiro, Brazil; 3 Universidade Federal Fluminense, Rio de Janeiro, Rio de Janeiro, Brazil; 4 Instituto Nacional de Ciência e Tecnologia de Biologia Estrutural e Bioimagem, Rio de Janeiro, Rio de Janeiro, Brazil; 5 Instituto Nacional de Cardiologia, Rio de Janeiro, Rio de Janeiro, Brazil; Universidad Autónoma de Yucatán, MEXICO

## Abstract

**Background:**

Chagas disease, caused by the protozoan *Trypanosoma cruzi* (*T*.*cruzi*), is a complex disease endemic in Central and South America. It has been gathering interest due to increases in non-vectorial forms of transmission, especially in developed countries. The objective of this work was to investigate if adipose tissue-derived mesenchymal stromal cells (ASC) can alter the course of the disease and attenuate pathology in a mouse model of chagasic cardiomyopathy.

**Methodology/Principal Findings:**

ASC were injected intraperitoneally at 3 days post-infection (dpi). Tracking by bioluminescence showed that cells remained in the abdominal cavity for up to 9 days after injection and most of them migrated to the abdominal or subcutaneous fat, an early parasite reservoir. ASC injection resulted in a significant reduction in blood parasitemia, which was followed by a decrease in cardiac tissue inflammation, parasitism and fibrosis at 30 dpi. At the same time point, analyses of cytokine release in cells isolated from the heart and exposed to *T*. *cruzi* antigens indicated an anti-inflammatory response in ASC-treated animals. In parallel, splenocytes exposed to the same antigens produced a pro-inflammatory response, which is important for the control of parasite replication, in placebo and ASC-treated groups. However, splenocytes from the ASC group released higher levels of IL-10. At 60 dpi, magnetic resonance imaging revealed that right ventricular (RV) dilation was prevented in ASC-treated mice.

**Conclusions/Significance:**

In conclusion, the injection of ASC early after *T*. *cruzi* infection prevents RV remodeling through the modulation of immune responses. Lymphoid organ response to the parasite promoted the control of parasite burden, while the heart, a target organ of Chagas disease, was protected from damage due to an improved control of inflammation in ASC-treated mice.

## Author Summary

Chagas disease is caused by the parasite *Trypanosoma cruzi*, which is transmitted by kissing bugs in endemic areas located in Central and South America. In its acute phase, the disease has unspecific and mild symptoms, whereas in the chronic phase infected individuals might develop severe cardiomyopathy. For many years, different cell types have been under investigation as alternatives for the treatment of several diseases. Mesenchymal stromal cells (MSC) are one of these cell types. As our understanding of MSC biology increases, their properties might be explored in different disease contexts. Since Chagas disease is characterized by intense inflammation and one of the key properties of MSC is the modulation of the immune system, our work investigated the effect of injecting MSC in a mouse model of Chagas disease. Our results show that MSC improved the control of parasite burden and cardiac inflammation, leading to the prevention of cardiac dilation.

## Introduction

Chagas disease, caused by the protozoan *Trypanosoma cruzi* (*T*. *cruzi*), is a complex systemic disease endemic in Central and South America. Classically transmitted by hematophagous triatomine insects, the disease has been receiving attention due to other forms of transmission such as blood transfusions, organ transplantation, ingestion of contaminated food or drinks and congenital transmission [1,2]. These vector-independent forms of contracting Chagas disease are the most common in developed countries, especially in North America, due to immigration of infected individuals from endemic areas [1,2]. Even though vector control initiatives have considerably reduced the number of infected individuals in endemic areas—in 1985 Latin America had ~17 million infected individuals, reduced to ~7.7 million in 2005 –estimates show that 20% of the Latin American population is at risk of infection [2]. In addition, there are 300,000 infected individuals living in the United States [1,3].

Chronic infection with *T*. *cruzi* can lead to three symptomatic forms: cardiac, digestive or cardiodigestive. The cardiac form is the most severe and frequent, affecting 20–30% of the infected individuals [1,2]. It can lead to a number of arrhythmic abnormalities, which make sudden death the most common cause of death in patients with chagasic cardiomyopathy (60–70% of deaths). Heart failure is a late manifestation of chagasic cardiomyopathy and is the second most frequent cause of death [2].

Antiparasitic drugs are not recommended for patients with end-stage heart failure as they will not resolve cardiac remodeling and have substantial side effects [3]. Cell therapy with bone marrow mononuclear cells has been tested as an alternative treatment in chagasic cardiomyopathy models and promising results were reported [4,5] (for a review see [6]). In this context, a randomized double-blinded placebo-controlled trial was conducted to test the efficacy of these cells in 183 patients with heart failure caused by Chagas disease [7]. Unfortunately, there was no improvement of cardiac function in these patients.

This does not mean, however, that cell therapy cannot be useful in Chagas disease. Adult stem cells are unlikely to be effective for end-stage heart failure, since they cannot directly generate new cardiac muscle [8–10]. Other stem cells, with proven cardiomyogenic potential, might be used for this purpose. Nonetheless, adult stem cells exert angiogenic, paracrine and immunomodulatory effects [10–13] that could target important pathogenic mechanisms in Chagas disease. There are four basic mechanisms thought to be responsible for chagasic cardiomyopathy: persistence of the parasite leading to chronic inflammation and myocardial damage, autoimmunity, dysautonomia and microvascular lesions [14]. Probably, the latter three are consequences of the myocardial damage induced by the parasite [1]. Mesenchymal stromal cells (MSC) act as sensors of inflammation mounting different responses depending on the microenvironment [13] and there is a strong crosstalk between MSC and the immune system [13,15]. In addition, these cells express several types of toll-like receptors (TLR) [15] that could directly recognize *T*. *cruzi*-derived pathogen associated molecular patterns (PAMP) [16]. Thus, when exposed to the microenvironment created by *T*. *cruzi*, MSC could respond, interact with the immune system and contribute to the control of myocardial damage.

The objective of this work was to investigate if adult adipose tissue-derived mesenchymal stromal cells (ASC) can alter the course of the disease and attenuate pathology in a mouse model of chagasic cardiomyopathy.

## Materials and Methods

### Ethics statement

All experiments were performed in conformity with the Guide for the Care and Use of Laboratory Animals (NIH) and were approved by the Committee on the Ethics of Animal Use of the Federal University of Rio de Janeiro under the number IBCCF 027 and 163/13.

### Animals

For cell isolation, 12 week-old C57/BL6-TgN(beta-act-EGFP) transgenic mice were used. These animals express the green fluorescent protein gene under the control of the β-actin promoter. For infection, 8–10 week-old CD1 mice weighing 25 to 30 g were used.

### ASC isolation

Animals were euthanized and subcutaneous inguinal fat pads were harvested. Tissue was minced into small pieces for enzymatic digestion with collagenase II 0.2% (Worthington) for 50 minutes at 37°C. Cells were plated in DMEM high-glucose supplemented with 20% fetal bovine serum (FBS, Gibco), 2 mM of L-Glutamine and 1% penicillin-streptomycin. Cultures were expanded and experiments were performed in passage 3.

### Colony forming units assay and population doubling time

Colony forming units (CFU) assay was performed to assess clonogenic potential of ASC. Cells were plated immediately after isolation in 6-well plates at a density of 3x10^3^ cells/well. The culture medium was the same used for expansion and cells were grown for 14 days. Then, cells were fixed in methanol for 5 minutes, stained with Giemsa and the number of colonies was manually counted.

For population doubling time (PDT), cells were plated in 35 mm gridded culture dishes (Nunc) (5x10^3^ cells/dish). Each grid had a known area of 4 mm^2^, allowing quantification of the number of cells per mm^2^. Four random grids were counted daily starting 24 hours after the cells were plated and henceforth until confluence was achieved. An exponential growth curve was built and transformed by plotting the y-axis in logarithm scale. Linear regression was performed and PDT was calculated from the reciprocal of the slope of the linear function obtained.

### Flow cytometry and cell differentiation

For flow cytometry, ASC were dissociated with trypsin-EDTA, washed once and ressuspended in PBS 0.5% BSA. Staining was done in a 100 μL volume per tube for 30 minutes at 4°C. Primary antibodies were diluted at 1:100 and were against the following antigens: CD90.2, Sca-1, CD105, CD73 and CD45 (all from eBioscience). Samples were acquired in BD FACSAria IIu and analyzed in FlowJo 9.4.10.

For osteogenic differentiation, cells were submitted to a 21-day culture protocol with DMEM supplemented with 10% FBS, penicillin-streptomycin, 10^−6^ μM of dexamethasone, 10 mM of β-glycerophosphate and 0.5 μM of ascorbic acid. After this period, cells were fixed and stained with Alizarin Red 1%. Adipogenic differentiation protocol was identical, except for β-glycerophosphate and ascorbic acid that were substituted for 200 μM of indomethacin, 10 μg/mL of human insulin and 0.5 mM of isobutyl-methylxanthine (all reagents from Sigma-Aldrich). Staining was performed with Oil Red O 0.5%.

### ASC transduction with luciferase 2

Lentiviral vector pMSCV.Luc2.T2A.Puro was constructed as previously described [17]. Lentiviral particles containing Luc2 and puromycin resistance genes were produced in HEK 293FT cells with pMSCV.Luc2.T2A.Puro vector and the accessory vectors pΔ8.9 and pHDM-VSV-G, using transfection reagent *FuGene 6* (Roche). After 48 and 72 hours of transfection, culture media containing lentiviral particles was filtered (0.45 μm, Corning Life Sciences) and centrifuged at 20000 x g.

ASC were cultivated with polybrene (8 μg/mL) (Sigma) and the lentiviral particles. After 24 hours of incubation, culture medium was replaced with the standard expansion medium. Approximately 48 hours after transduction, 0.2 μg/mL of puromycin was added to the medium. Cells were selected during seven days and then expanded for bioluminescence imaging assay.

### Tracking by bioluminescence

For *in vitro* studies, transduced cells were plated in a 24-well plate at different concentrations: 10^3^, 2x10^3^, 5x10^3^, 8x10^3^, 10^4^, 2x10^4^, 5x10^4^, 8x10^4^, and 10^5^ cells/well. D-Luciferin (150 μg/mL) (Promega Corporation) was added to the culture medium. The plate was immediately positioned in the IVIS Lumina Imaging System (Caliper Life Sciences) and images were acquired after a 10-second exposure period.

For *in vivo* studies, mice received D-Luciferin (150 mg/kg) intraperitoneally. Ten minutes after injection, they were anesthetized with isoflurane gas and placed in the IVIS Lumina Imaging System. Image acquisitions were performed from day 1 to 9 after the injection of transduced cells. Exposure time never exceeded 5 minutes, to avoid false-negative results.

For *ex vivo* studies, mice received D-Luciferin (150 mg/kg) intraperitoneally. Ten minutes after injection, they were euthanized; the organs were removed and placed in a 24-well plate with PBS. Images were acquired on days 1, 2 and 4 after cell injection.

Results were analyzed in Living Image Software 3.2 (Caliper Life Sciences).

### Infection with T. cruzi and cell transplantation

CD1 mice were infected with an intraperitoneal (IP) injection of 3x10^4^ trypomastigotes of the Brazil strain of *T*. *cruzi*, which belongs to the discrete typing unit TcI [18,19]. To evaluate parasitemia, tail vein blood was collected from infected animals every other day from the 5^th^ to the 32^th^ day post-infection (dpi). Blood samples of 10 μL were diluted in ammonium chloride 0.85% and parasites were counted in a hemocytometer.

ASC were dissociated from culture flasks with trypsin-EDTA and counted in a hemocytometer. One million cells were diluted in 200 μL of PBS and injected intraperitoneally on the 3^rd^ dpi in the cell-treated group. Placebo group was submitted to an identical procedure receiving only PBS. Animals were randomly assigned to the experimental groups. The study design is shown in S1 Fig.

### Cytokine analyses

The presence of IFN-γ, TNF-α, transforming growth factor-β (TGF-β), interleukin-2 (IL-2), IL-6 and IL-10 was investigated in the supernatants of spleen and heart cultures obtained at 30 dpi from infected mice treated either with placebo or ASC. Briefly, the spleen was removed and mechanically disintegrated. Red blood cells were removed with lysis buffer (Sigma) and cells were plated in RPMI 1640 with 10% FBS and antibiotics. For the isolation of heart cells, blood was thoroughly removed and the tissue was minced and digested with collagenase II 0.2%. Cells were plated under the same conditions as the splenocytes. The cultures were incubated for 48 hours in the presence of trypomastigote lysates, which were obtained through repeated freezing and thawing of the parasites. After 48 hours, the supernatants were collected and analyzed by ELISA (R&D Systems) or Cytokine Bead Array (CBA, BD Biosciences). ELISA and CBA experiments were performed with at least two technical replicates. The same cytokines, except for TGF-β, were also analyzed in the serum of non-infected, placebo and ASC groups at 30 dpi.

### In vitro infection assay

Mouse peritoneal macrophages were collected and plated in DMEM with 10% of heat inactivated FBS for 3 hours. Then, trypomastigotes were added to the cultures at a proportion of 10 parasites to 1 cell for 5 hours. After this period, one sample was washed and fixed with paraformaldehyde 4% to provide baseline infection counts and the other samples were thoroughly washed to remove trypomastigotes that had not invaded macrophages. Mitotically inactivated ASC (30 Gy of γ-radiation) were added at a proportion of 1 ASC to 1 macrophage for 72 hours. Mitotic inactivation was necessary to prevent overgrowth and allow for appropriate counting of amastigotes in infected macrophages. After 72 hours, samples were fixed and stained with DAPI. The number of amastigotes per macrophage was estimated by counting under a fluorescence microscope. The experiment was performed in duplicates and at least 120 infected cells were counted.

### Histology

Histology was performed at 30 dpi. Hearts were washed in PBS and fixed in paraformaldehyde 4% for 24 hours. Subsequently, samples were paraffin embedded and 8 μm slices were obtained. Slides were stained with Hematoxilin & Eosin (H&E) and Sirius Red. Inflammation was quantified in 30 random fields by counting the number of inflammatory foci (aggregates containing more than 7 cells in a 5 μm^2^ area). The number of amastigote nests was quantified by direct counting of 20 random fields. Fibrosis was also quantified in 20 random fields as a percentage of the total area of cardiac tissue using Image-Pro Plus 5.0 software.

### Echocardiography and magnetic resonance imaging

Functional analysis was performed at 60 dpi in cell-treated and placebo groups. For echocardiogram exams, mice were anesthetized with 1.5% isoflurane in O_2_ and the thoracic region was shaved. A small gel standoff was placed between the chest and a 30 MHz ultrasound scanhead connected to the Vevo 770 Imaging System (VisualSonics). Heart rate and body temperature were monitored during all exams. Geometric analyses were made using B-mode images and left ventricular (LV) ejection fraction (EF) was calculated by Simpson’s rule. Right ventricular (RV) area was also obtained in B-mode using a short axis view at the level of the papillary muscles.

*In vivo* magnetic resonance cardiac imaging (MRI) was performed on a 7.0T horizontal-bore scanner (Varian Medical Systems) under inhalation anesthesia applied by nose cone (1.5% isoflurane in O_2_). High-resolution bright-blood MRI experiments were conducted using an ECG-triggered fast low-angle shot (FLASH) gradient-echo (GE) pulse sequence tailored for murine imaging. Hearts were imaged from the base to the apex by a stack of two-dimensional images. The electrocardiographic gating was optimized with two subcutaneous precordial leads with respiratory motion and body temperature monitors (SA Instruments). The scanning parameters were optimized for the signal-to-noise ratio (SNR) as follows: flip angle = 30°, echo time (TE) = 1.9 ms, repetition time (TR)≅ R-R interval, radiofrequency (RF) pulse width = 1.0 ms, number of averages = 8 and 15 frames per heart cycle were obtained. All images were acquired with a field of view (FOV) of 30x30mm and data matrix of 128x128mm to yield an in-plane resolution of 234. Total scan time was in the range of 25 minutes. Each imaging protocol resulted in five to eight 1 mm thick short-axis images covering the whole heart from apex to base with no gap between slices. The data were analyzed with OsiriX Imaging software. Ventricular slice volumes were determined from end-diastolic and end-systolic images by multiplication of compartment area and slice thickness. End-diastolic volume (EDV) and end-systolic volume (ESV) were calculated as the sum of all slices and EF was calculated by Simpson’s rule for both the left and right ventricles.

### Statistical analyses

Data are presented as mean ± SEM. Cardiac function, cytokine data and the infection assay were analyzed using one-way analysis of variance (ANOVA) with Bonferroni’s multiple comparison test. The area under the parasitemia curve (AUC) was calculated for each animal in both experimental groups. AUC and histology data were analyzed using Student’s t-test. PDT was analyzed using linear regression and the *in vitro* luminescence data was analyzed using Pearson correlation. All analyses were performed with GraphPad Prism 6.0 software and p<0.05 was considered significant.

## Results and Discussion

### Characterization of mouse adipose tissue-derived mesenchymal cells

Interest in Chagas disease has been increasing as new forms of transmission arise, especially in non-endemic areas [3]. Since its discovery, over one century ago, the pathogenesis of the disease remains elusive. Upon infection, the parasite starts to replicate, eliciting an inflammatory response in host tissues [20], which is essential to maintain parasite levels under control. In this context, it has been demonstrated that immunosuppression in transplanted patients may lead to reactivation of the disease [21]. A balance between parasite replication and inflammation is usually achieved, leading to parasite persistence and, consequently, to the chronic stages of Chagas disease [20].

In this work, we show that administration of adipose tissue-derived mesenchymal stromal cells soon after infection with *T*. *cruzi* modifies the systemic response to the parasite in mice.

**Fig 1:**
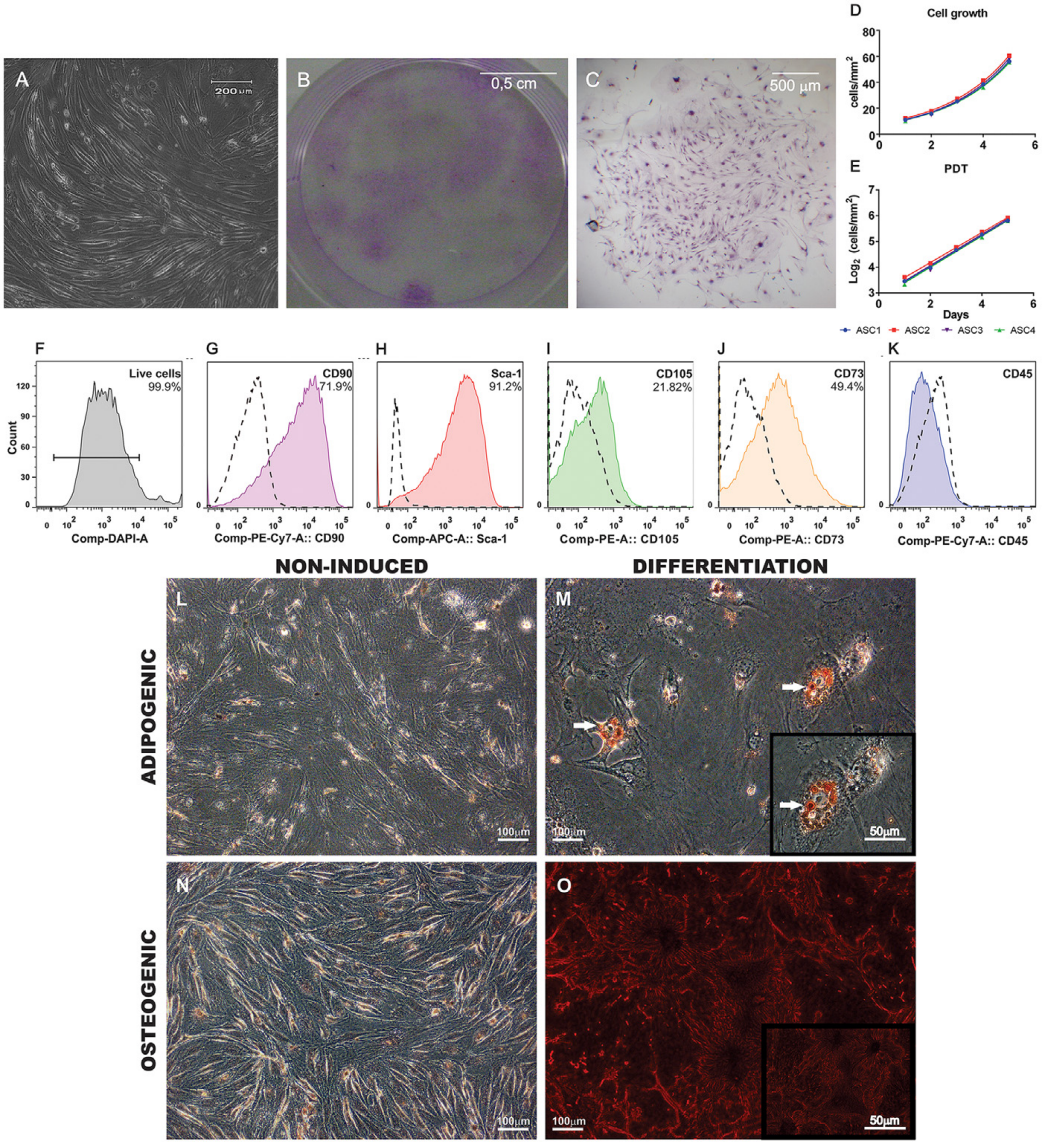
Characterization of ASC. (A) ASC were adherent to culture flaks and exhibited a fibroblast-like spindle-shaped morphology. (B) Macroscopic view of CFU-F assay showing colonies of ASC stained by Giemsa. (C) Microscopic view exemplifying one colony of ASC also stained by Giemsa. Growth of ASC in culture was exponential (D) and the curves were converted to logarithmic scale (E) to calculate population doubling time (PDT) (n = 4 in all experiments). (F-K) Histograms showing the immunophenotype of ASC by flow cytometry. (F) Dead cells were excluded from the analysis by DAPI staining. (G-K) Dashed lines represent isotype controls and the percentage of positive events is shown on the right upper quadrant. Cells were predominantly positive for CD90.2 (G) and Sca-1 (H). Expression of CD105 (I) and CD73 (J) was lower. No hematopoietic contaminants were observed since cells were negative for CD45 (K). (L-O) Differentiation of ASC. (L and N) Negative controls. Cells were cultured for 21 days in standard expansion culture medium. (M) ASC cultured in adipogenic conditions exhibited lipid vacuoles in their cytoplasm shown by Oil Red O staining (white arrows). (O) Cells cultured in osteogenic conditions exhibited calcium deposits in the extracellular matrix stained by Alizarin Red. Inserts show higher magnification images of M and O.

ASC were isolated from subcutaneous inguinal fat pads, expanded and characterized. Cells were adherent to plastic culture flasks and displayed a spindle-shaped morphology, as expected for this cell type (Fig 1A). When grown in CFU-F conditions, ASC generated 32.25 ± 1.26 clones for every 3x10^3^ cells plated. Macroscopic and microscopic images of the colonies can be seen in Fig 1B and 1C respectively. Cells presented an exponential growth (Fig 1D) and population doubling time was 40.15 ± 0.37 hours.

Flow cytometry revealed a population predominantly positive for CD90.2 and Sca-1, two molecules frequently present in mouse mesenchymal cells (Fig 1G and 1H respectively). Expression of CD105 and CD73 was lower, while no CD45 positive cells were found in our samples, indicating that no hematopoietic contaminants were present (Fig 1I, 1J and 1K). Additionally, upon induction, ASC differentiated into adipogenic and osteogenic lineages (Fig 1M and 1O).

### Distribution of ASC after cell transplantation

**Fig 2:**
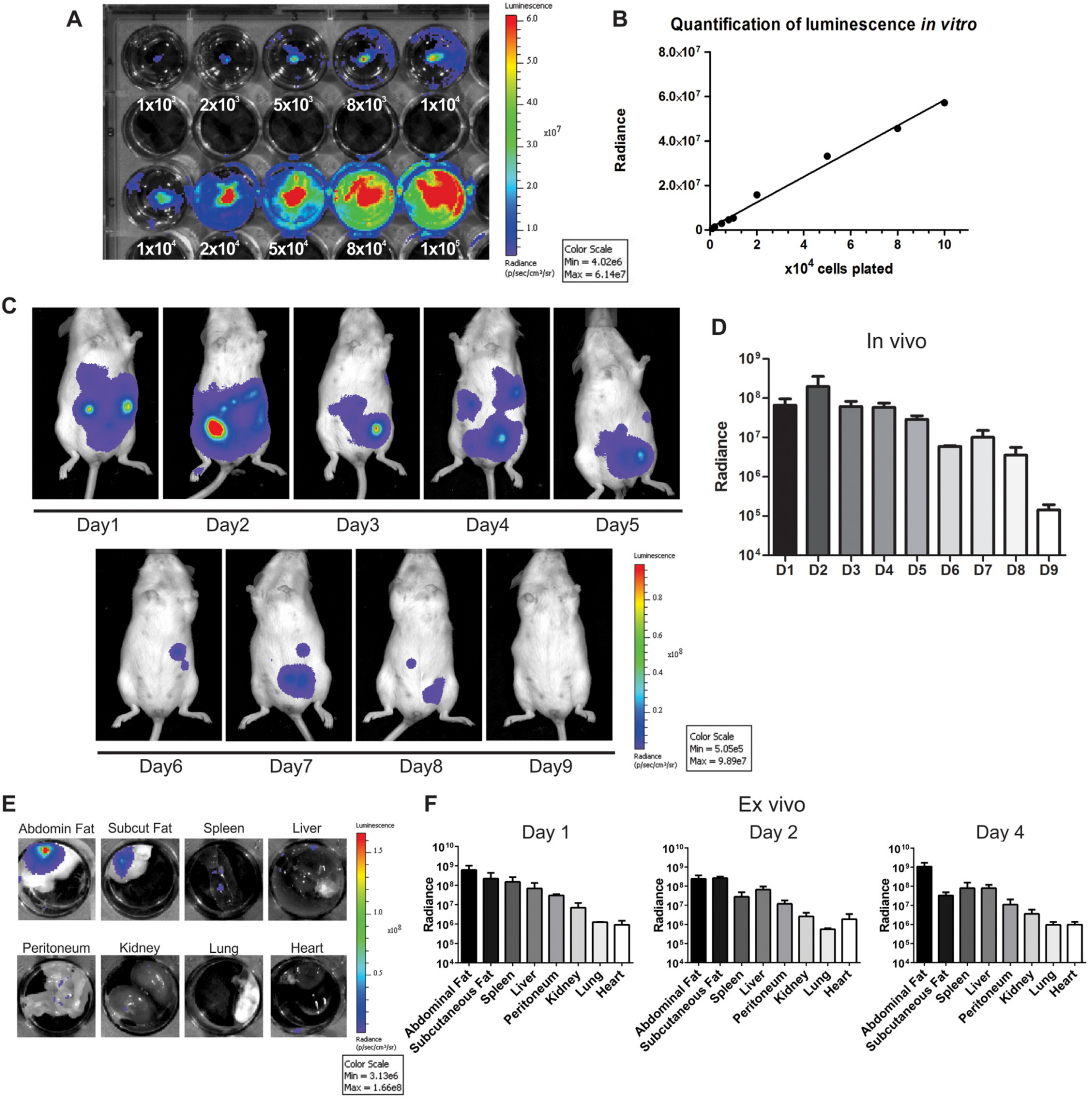
Cell tracking by bioluminescence. (A) Evaluation of luminescent signal intensity in ASC transduced with the Luciferase 2 gene *in vitro*. Numbers shown below the wells indicate the amount of cells. (B) The signal increased with cell numbers in a linear fashion (R^2^ = 0.9914). *In vivo* images (C) and quantification (D) (n = 3) of the luminescent signal showed that cells remained in the abdominal region and that there was a progressive decrease in radiance with time. The signal disappeared within 9 days of cell injection. *Ex vivo* images (E) and quantification (F) (n = 3 per day) of the luminescent signal demonstrated that the majority of the cells migrated to the abdominal or subcutaneous fat. The signal was lower in other abdominal organs, such as the spleen and liver, and virtually absent in thoracic organs such as the heart and lungs. Quantification data in D and F are shown in logarithmic scale.

ASC were transduced with the Luciferase 2 gene and the luminescent signal was evaluated *in vitro*. There was a strong linear correlation between cell number and the luminescent signal (R^2^ = 0.9914, Fig 2B), demonstrating that this approach could be reliably used to track and quantify cell distribution *in vivo*.

One million ASC were injected intraperitoneally in *T*. *cruzi* infected mice at 3 dpi. The use of intraperitoneal injections was not our original design for the study, but mice did not tolerate intravenous injection of ASC due to pulmonary embolism. The luminescent signal indicated that the cells remained in the abdominal cavity from day 1 up to day 9 after injection (Fig 2C and 2D). In addition, the signal was progressively reduced over time, suggesting that ASC engraftment was not permanent. *Ex vivo* analysis showed that the majority of the cells migrated from the peritoneal cavity to the abdominal and subcutaneous fat and remained there at least until day 4 after injection (Fig 2E and 2F). Luminescence was less intense in the liver, spleen and peritoneum. The heart and lungs exhibited signal intensity close to the lower limit of detection of our imaging system.

ASC homing to the adipose tissue of infected animals could be explained by the fact that cells were attracted to their niche or because the adipose tissue acts as a parasite reservoir during acute infection. It is well known that *T*. *cruzi* has a strong adipose tissue tropism, as has been demonstrated by several independent investigators [22–25]. Recent work by Tanowitz’s group has shown that parasitism at 15 days post infection was higher in the adipose tissue than in the heart and spleen of mice infected with the Brazil strain of *T*. *cruzi* [25]. In addition, inflammatory signals, which have been demonstrated to induce recruitment of MSC [26], are also increased in adipose tissue after infection [25]. Hence, ASC homing to an early parasite reservoir could suggest that cells were attracted by the inflammatory process.

### ASC reduce parasitism, inflammation and fibrosis in the heart of chagasic mice

**Fig 3:**
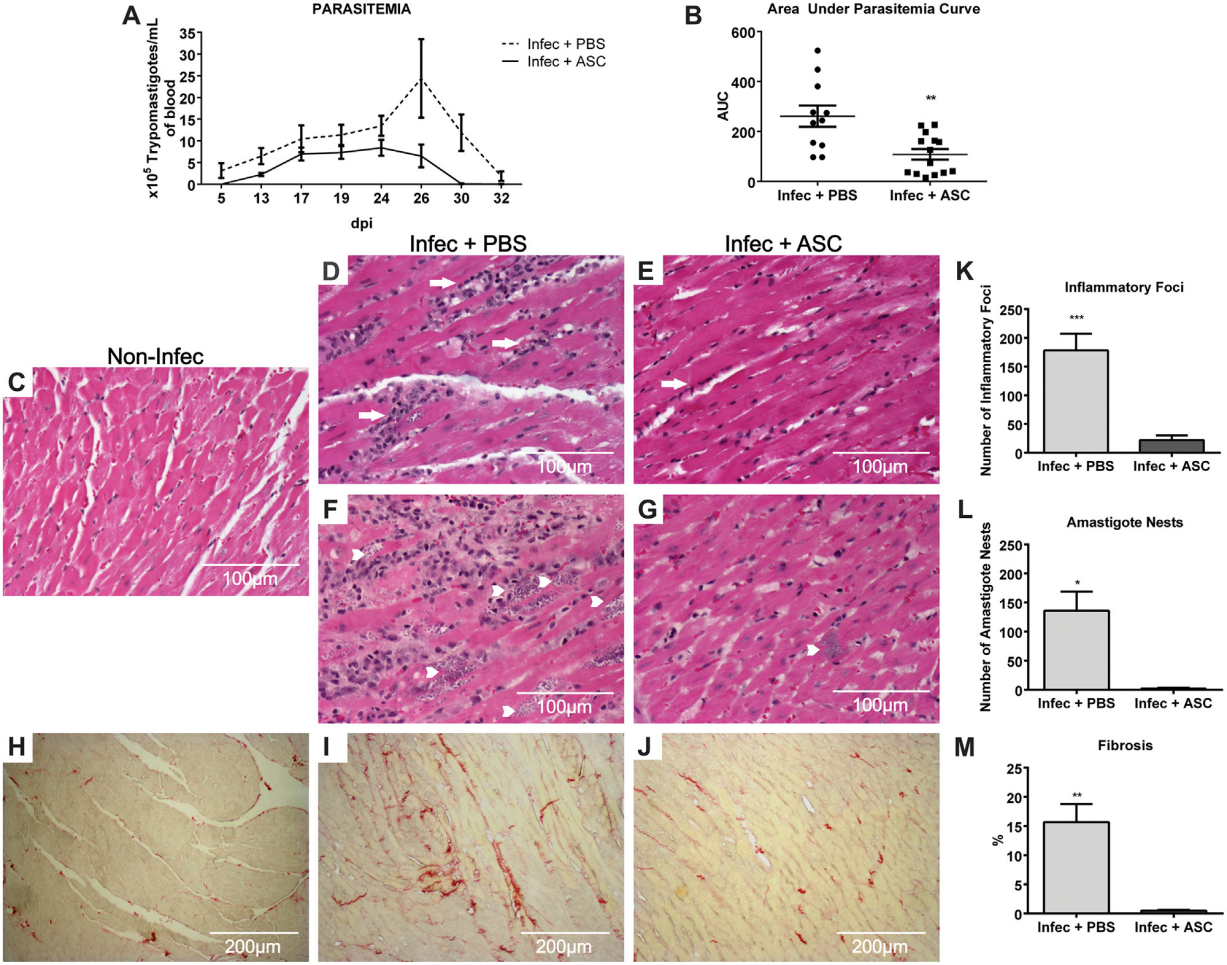
Parasitism, inflammation and fibrosis in infected mice. (A) Number of parasites (x10^5^) per mL of blood as days after infection progress. The area under the parasitemia curve was calculated for each animal and plotted in (B). ASC-treated animals (n = 14) had a significantly lower AUC when compared to placebo (n = 11) (**p = 0.0022). (C-G) H&E and (H-J) Sirius Red staining of heart tissue sections. (C) Non-infected animals showed normal myocardial fibers. Infection with *T*. *cruzi* caused a significant increase in the number of mononuclear inflammatory foci (arrows) (D) and amastigote nests (arrow heads) (F). Therapy with ASC prevented myocardial inflammation (E) and reduced the number of amastigote nests in the heart (G). Additionally, fibrosis was also increased in infected animals treated with placebo (I) when compared to non-infected mice (H), as shown by collagen fibers stained in red. Treatment with ASC reduced fibrosis in infected animals (J). Quantification of inflammation (K) (n = 7, ***p = 0.0002), amastigote nests (L) (n = 3, *p = 0.015) and fibrosis (M) (n = 3, **p = 0.0083) compared to Infec+ASC, Student’s t test.

After cell transplantation, the number of parasites per mL of blood was quantified. The peak of parasitemia was reached between 24 and 26 dpi (Fig 3A) and the areas under the parasitemia curves were significantly smaller in ASC-treated when compared to placebo-treated animals (108.3 ± 21.20 vs 261.3 ± 42.44 respectively) (Fig 3B). This effect on parasitemia was unexpected. It suggests ASC influenced the control of parasite burden either directly or indirectly, by modulating the immune response. *T*. *cruzi* replication control is an elusive subject. On one hand, it is known to be dependent on a T helper 1 (Th1) type response, as the absence of cytokines such as TNF-α and IFN-γ (or its receptors) leads to excessive parasite replication [20,27]. On the other hand, the Th1 response can be harmful in chronically infected patients, in which high levels of these cytokines have been correlated to cardiac dysfunction [28]. Hence, the nature of the immune response and the degree of parasite persistence are under a delicate equilibrium that determines the progression of pathology. This balance seems to have shifted when ASC were injected early after infection, resulting in a more efficient control of parasitemia.

To further explore the effects promoted by ASC, inflammation, parasitism and fibrosis were analyzed in the hearts of infected mice at 30 dpi (Fig 3). There was an 8-fold decrease in the number of inflammatory foci in cell-treated compared to placebo-treated mice (22.00 ± 3.05 and 178.10 ± 28.96 respectively) (Fig 3K). Moreover, the number of amastigote nests in cardiac tissue was much lower in ASC-treated animals (2.00 ± 1.00) when compared to placebo (136.00 ± 32.75) (Fig 3L). Finally, ASC also prevented the accumulation of collagen fibers in the heart (cell-treated 0.52 ± 0.05% vs placebo 15.65 ± 3.12%) (Fig 3M).

### ASC modify cytokine production in mice infected with T. cruzi

**Fig 4:**
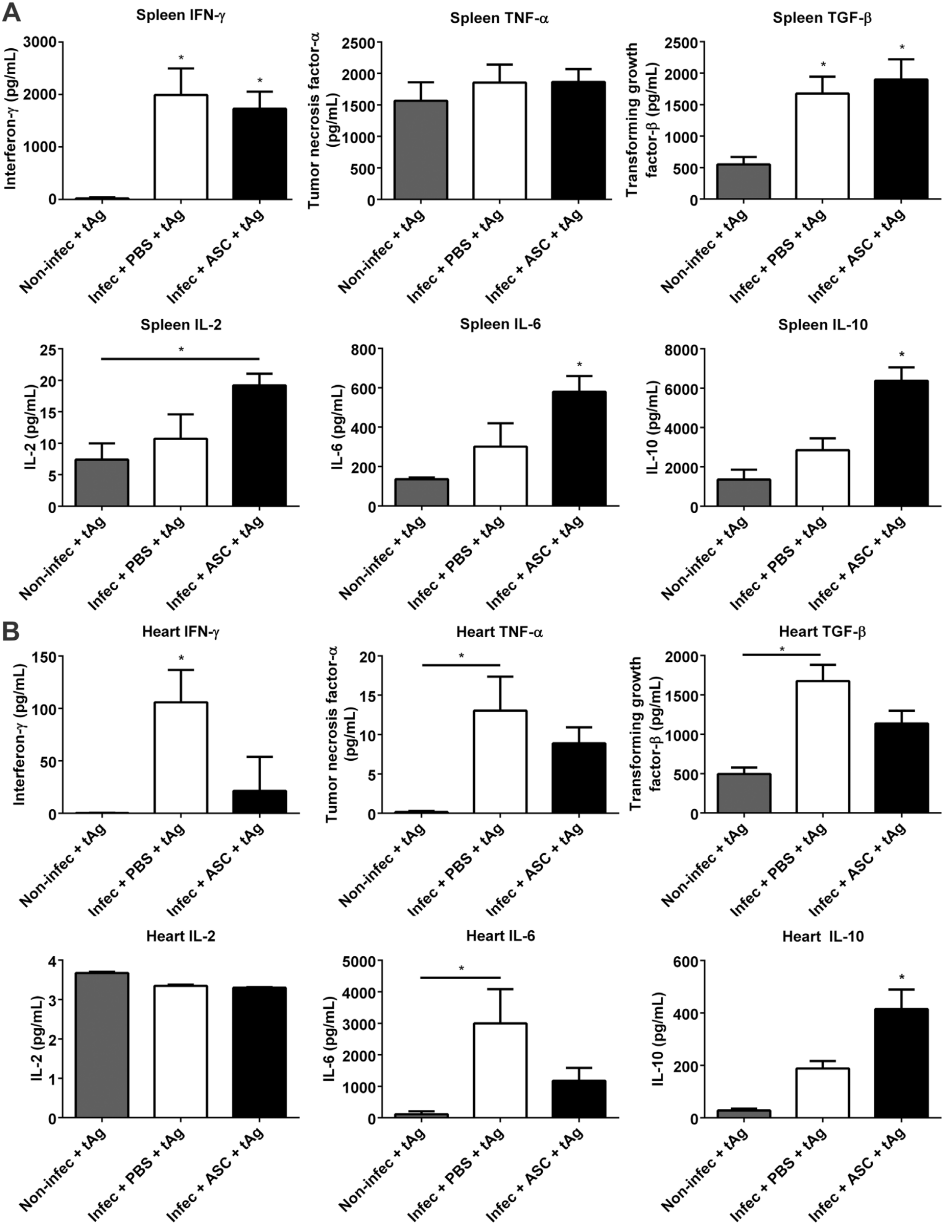
Cytokine release in non-infected and infected mice in response to *T*. *cruzi* antigens (tAg). (A) Splenocytes from infected animals released significantly higher levels of IFN-γ and TGF-β when compared to non-infected controls. No differences were found between placebo and ASC-treated groups in IFN-γ, TNF-α and TGF-β. IL-2 levels were increased in ASC-treated samples when compared to non-infected controls. IL-6 and IL-10 were higher in the ASC group when compared to placebo and non-infected groups. (B) When stimulated with tAg, cells derived from hearts treated with ASC released less IFN-γ and more IL-10 in comparison to placebo-treated animals. Levels of TNF-α, TGF-β and IL-6 were significantly increased in the placebo group when compared to non-infected controls, while the ASC group had intermediate levels of these cytokines. IL-2 release was low and similar between the three experimental groups (*p<0.05, numbers of samples in each group were at least non-infec: 4, placebo: 5, ASC: 5).

In order to investigate possible mechanisms linked to the decrease in inflammation promoted by ASC, we analyzed the profile of organ-specific cytokine production *in vitro* at 30 dpi in infected mice treated with ASC or placebo, as well as in non-infected controls. When cultured with *T*. *cruzi* antigens, the release of IFN-γ and TGF-β was significantly higher in splenocytes obtained from infected animals when compared to non-infected controls (Fig 4A). In addition, an increase in IFN-γ was also observed in the serum of infected animals (S2A Fig). No differences were found in TNF-α release by splenocytes from infected animals when compared to non-infected controls (Fig 4A). However, there was a significant increase in serum TNF-α levels in infected animals (S2B Fig). These results suggest that the peripheral immune response displayed a sustained inflammatory phenotype, which could contribute to the control of blood parasitemia. Placebo and ASC-treated groups displayed similar levels of IFN-γ, TGF-β and TNF-α in splenocytes (Fig 4A). On the other hand, splenocytes derived from the ASC-treated animals released significantly higher levels of IL-10 when compared to the placebo group (Fig 4A), revealing that ASC treatment strengthened an anti-inflammatory arm that could contribute to the attenuation of tissue damage provoked by sustained inflammation in the heart. Moreover, IL-6 release was higher in ASC group when compared to placebo-treated animals (Fig 4A). The serum levels of IL-2, IL-6 and IL-10 were at the lower limit of detection and were similar in the three experimental groups. Taken together, these results indicate a pro-inflammatory Th1-type response to the parasite in infected animals, which was also reflected in the serum as IFN-γ and TNF-α were elevated in placebo and ASC-treated mice. Notably, although IL-10 is anti-inflammatory and characteristic of Th2 type responses, it has been demonstrated that Th1 cells can also produce IL-10, leading to an improved control of immune responses against parasites [29].

In contrast, heart cells displayed a different pattern of cytokine release when re-called with *T*. *cruzi* antigens *in vitro*. In ASC-treated animals, IFN-γ was reduced to the levels of non-infected animals, while it remained elevated in the placebo group (Fig 4B). In agreement with an inflammatory environment in the heart, TNF-α and IL-6 were higher in the placebo group when compared to non-infected animals, while the ASC group showed decreased levels of those cytokines (Fig 4B). No differences were found in IL-2 levels between the three experimental groups. Importantly, IL-10 release was increased in ASC-treated animals when compared to placebo and non-infected mice (Fig 4B). These results indicate that even though IFN-γ producing cells are likely similar in number and/or degree of activation in the secondary lymphoid organs (i.e. spleen) of infected animals, they seem less competent to reach the heart when infected mice are treated with ASC. In addition, the higher levels of heart IL-10 suggest that, in ASC-treated mice, anti-inflammatory cells accumulate in higher proportion at the site of tissue parasitism, potentially contributing to attenuate and/or prevent tissue damage. Importantly, IL-10 derived from CD8^+^ T lymphocytes, either double IFN-γ/IL-10 or single IL-10 producing T cells, was found to limit parasite burden and protect mice from acute myocarditis in a mouse model of Chagas disease [30]. Moreover, IL-10^-/-^ mice failed to control blood parasitemia and mortality was 100%, indicating that IL-10 has a protective role in acute *T*. *cruzi* infection [30]. Increased IL-10 in the hearts of ASC-treated animals could be ultimately responsible for the reduction in the number of nests and inflammatory foci. Lower heart parasitism could also result in diminished chemokine release, controlling the degree and pattern of cellular recruitment. Indeed, parasite factors are known to influence the degree of chemokine release by macrophages [31]. It remains to be investigated the nature of the cellular populations contributing to the pro- and anti-inflammatory phenotypes in the heart of ASC-treated animals, as well as their homing receptors.

These data on cytokine release are consistent with our previous findings on heart inflammation. The reduction of IFN-γ levels in the heart combined with the concomitant increase in IL-10 most likely contributed to the control of inflammation, resulting in higher tissue preservation. The diminished parasite load could have further contributed to the reduction in local inflammation and scaring of cardiac tissue.

To investigate if the effects promoted by ASC could be attributed to a direct interference with parasite replication, peritoneal macrophages were infected and co-cultured with irradiated ASC. After 72 hours, the number of amastigotes per cell was significantly increased in infected macrophages when compared to baseline (S3 Fig). However, the presence of ASC interfered with parasite replication/development, resulting in reduced numbers of amastigotes per macrophage at the 72-hour time point. Hence, it is possible that ASC can further contribute to the control of parasitism *in vivo* by acting directly in the microbicidal potential of macrophages. However, we cannot exclude that there is also an indirect effect on parasite replication through the modulation of innate responses. In fact, when analyzing the peritoneal lavage of infected animals treated with ASC at 7 dpi (or 4 days post-treatment), IFN-γ levels were slightly increased when compared to placebo-treated mice (S4 Fig).

### ASC prevent right ventricular dilation in chagasic mice

**Fig 5:**
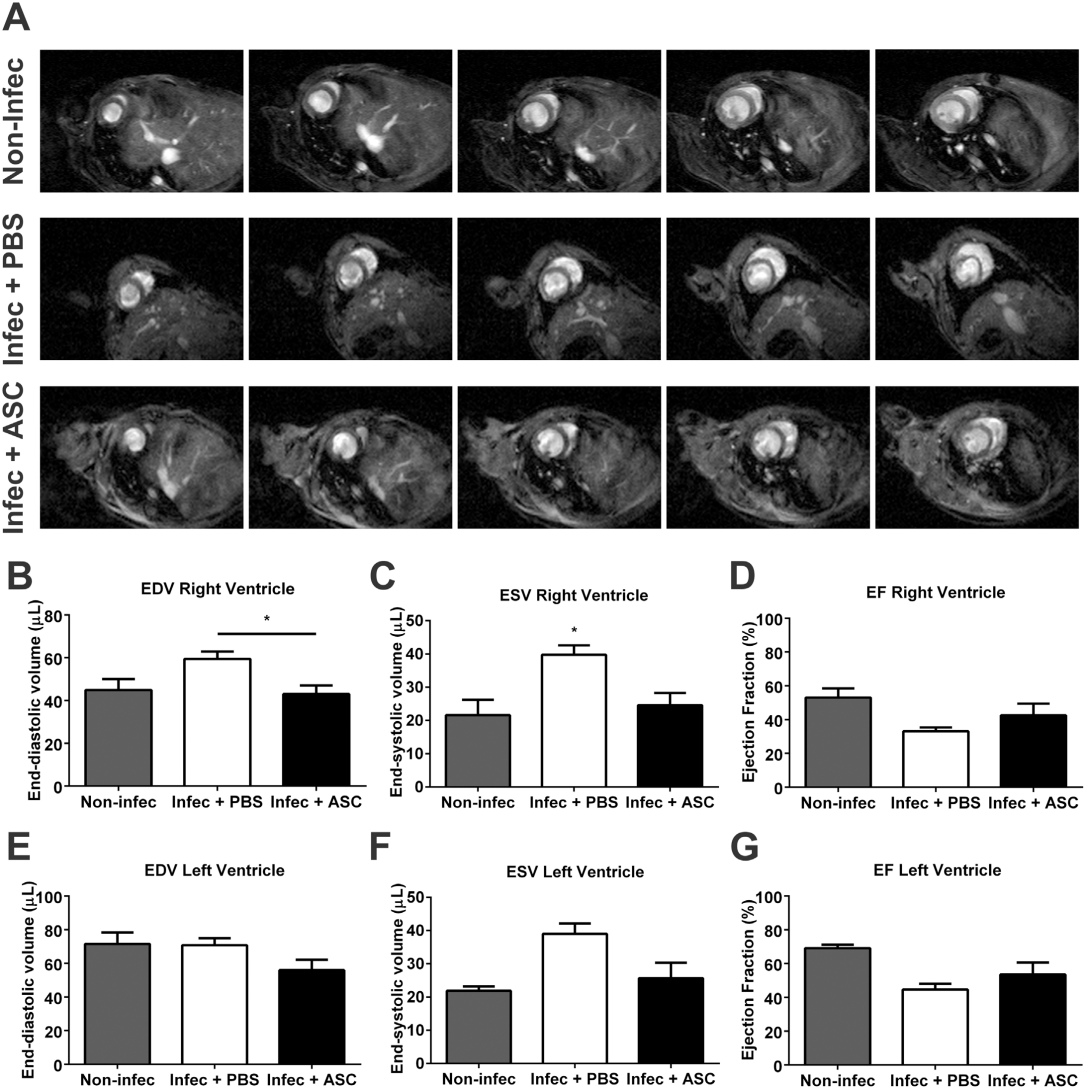
Magnetic resonance imaging of chagasic mice. (A) Representative magnetic resonance images in diastole. A sequence of images from the same animal is shown in each line. Observe the enlargement of the right ventricle (RV) in the placebo group. (B) RV end-diastolic volume (EDV) was significantly higher in placebo-treated when compared to ASC-treated animals. (C) RV end-systolic volume (ESV) was significantly higher in placebo-treated when compared to ASC-treated and non-infected mice. No differences were found in RV ejection fraction (EF) (D) or in left ventricular EDV (E), ESV (F) or EF (G) (*p<0.05, non-infec n = 3, placebo n = 6, ASC n = 6 for all parameters).

To determine if ASC injection would lead to an improvement in cardiac remodeling, mice were submitted to MRI (Fig 5A) and echocardiography at 60 dpi. MRI revealed that placebo-treated animals had a significant increase in right ventricular EDV when compared to ASC-treated mice (59.41 ± 3.52 vs 43.06 ± 3.97 μL respectively), indicating cavity dilation (Fig 5B). In addition, right ventricular ESV was also increased in the placebo group when compared to the ASC group (39.77 ± 2.83 vs 24.62 ± 3.66 μL respectively), suggesting that right systolic function was compromised (Fig 5C). Nonetheless, RV ejection fraction was not statistically altered between experimental groups (placebo 33.09 ± 2.29 vs ASC 42.57 ± 6.85% respectively), although there is a tendency towards a reduction in placebo-treated mice (Fig 5D).

As expected for this model [4], we did not observe significant changes in LV EDV, ESV or ejection fraction, although there is also a tendency towards an increase in ESV in the placebo group (Fig 5E, 5F and 5G).

Echocardiogram at 60 dpi revealed no differences in RV area when comparing placebo and cell-treated mice (S5A Fig). A similar analysis done by MRI showed a significant increase in RV area in the placebo group (S5B Fig), suggesting that echocardiography is not sensitive enough to detect RV alterations at this time point in this model. In addition, no differences were detected in LV ejection fraction by echocardiography (S5C Fig).

It is important that ASC effect on inflammation, parasitism and cytokine release was accompanied by a reduction in RV dilation, as this suggests an impact of our immune-related results in cardiac remodeling. However, although the mouse has been widely used to model Chagas disease in immunological studies, when considering left ventricular dysfunction, mice do not seem to reproduce human disease and this is a limitation for all studies that attempt to evaluate therapies for chagasic cardiomyopathy. Hence, the relevance our findings for the clinical translation of cell therapy in Chagas disease is limited and studies on larger animal models are being conducted to reach a definitive conclusion and address if this will be therapeutically applicable. The use of ASC at 3 dpi is unlikely in the clinical setting and it is possible that they might not induce the same effects if cells are injected at later stages, when the immune response against the parasite has been mounted. In this regard, three studies have tested MSC in Chagas disease models in mice, although the results are not directly comparable to ours due to differences in study design. Jasmin and colleagues showed that after intravenous injection of bone marrow-derived MSC the majority of the cells are trapped in the lungs, liver or spleen [32,33]. In addition, therapy was given at 30 dpi [32] or 60 dpi [33], time points in which the peak of parasitemia has already receded. Importantly, in regard to the time window for MSC therapy after infection, these studies also indicated that cell therapy led to a reduction in RV area at 30 dpi [32] and diameter at 60 dpi [33], but inflammation and fibrosis were not evaluated. Another study, by Larocca and co-workers, treated mice with ASC in the chronic phase, at 6 months post infection [34]. As reported for bone marrow mononuclear cells, they observed a reduction in inflammation and fibrosis, but there was no impact on the frequency of arrhythmias or tolerance to exercise. Besides analyzing the chronic phase of the disease and using a different mouse and *T*. *cruzi* strain, this study lacks mechanical cardiac function analyses, cell tracking, ASC characterization, as well as mechanistic data to explain the anti-inflammatory effects of ASC chagasic cardiomyopathy.

In conclusion, our work demonstrates that the injection of ASC early after *T*. *cruzi* infection prevents RV remodeling through the reduction of blood parasitemia, heart inflammation and fibrosis, as well as through alterations in cytokine release in response to parasite antigens. The mechanism responsible for these effects still needs to be further investigated, but it is likely that ASC participate in the modulation of innate and adaptive immune responses, improving the control of parasite replication. This leads to lower parasitism in heart, reduces myocardial damage and improves control of the inflammatory process. It will be interesting to see if these cells are effective in association with antitrypanosomal drugs, as this would target two of the most important pathologic mechanisms involved in Chagas disease: parasite persistence and heart tissue inflammation.

## Supporting Information

S1 Fig Experimental design. (TIF)

S2 Fig Concentration of IFN-γ (A) and TNF-α (B) in serum samples obtained from infected animals treated with placebo (n = 7) or ASC (n = 7), as well as non-infected controls (n = 5). Both cytokines were increased in the infected groups when compared to non-infected animals (*p<0.05). No differences were found between placebo and ASC groups.
(TIF)

S3 Fig *In vitro* infection assay on macrophages co-cultured with ASC. After 72 hours, there was an increase in the number of amastigotes per macrophage when compared to baseline (Infec 5h). However, co-culture with ASC reduced the number of amastigotes per cell when compared to isolated macrophages in the same time point (*p<0.05 compared to Infec 5h, ^#^p<0.05 compared to Infec + ASC 72h).
(TIF)

S4 Fig Interferon-γ levels on peritoneal lavage at 7 dpi. Flow cytometry histograms showing fluorescence intensity of interferon-γ in placebo (blue) and ASC-treated mice (green), as well as non-infected controls (red). The negative control is shown in purple and each histogram represents one animal. The ASC group showed a slight increase in fluorescence intensity of interferon-γ when compared to the placebo group and to non-infected mice, which were similar to the negative control.
(TIF)

S5 Fig Echocardiography of chagasic mice. (A) No differences were found in right ventricular area when comparing non-infected, placebo-treated and ASC-treated mice. However, when the same measurement was done by MRI, there was a significant increase in RV area in placebo-treated when compared to cell-treated animals. (C) LV ejection fraction measured by echocardiography was not different among the three experimental groups.
(TIF)
