# Supplementary figures and images for: Adipose Tissue-Derived Mesenchymal Stromal Cells Protect Mice Infected with Trypanosoma cruzi from Cardiac Damage through Modulation of Anti-parasite Immunity
Source: PLoS Negl Trop Dis. 2015 Aug 6;9(8):e0003945. doi: 10.1371/journal.pntd.0003945 (PMC4527728; doi:10.1371/journal.pntd.0003945)

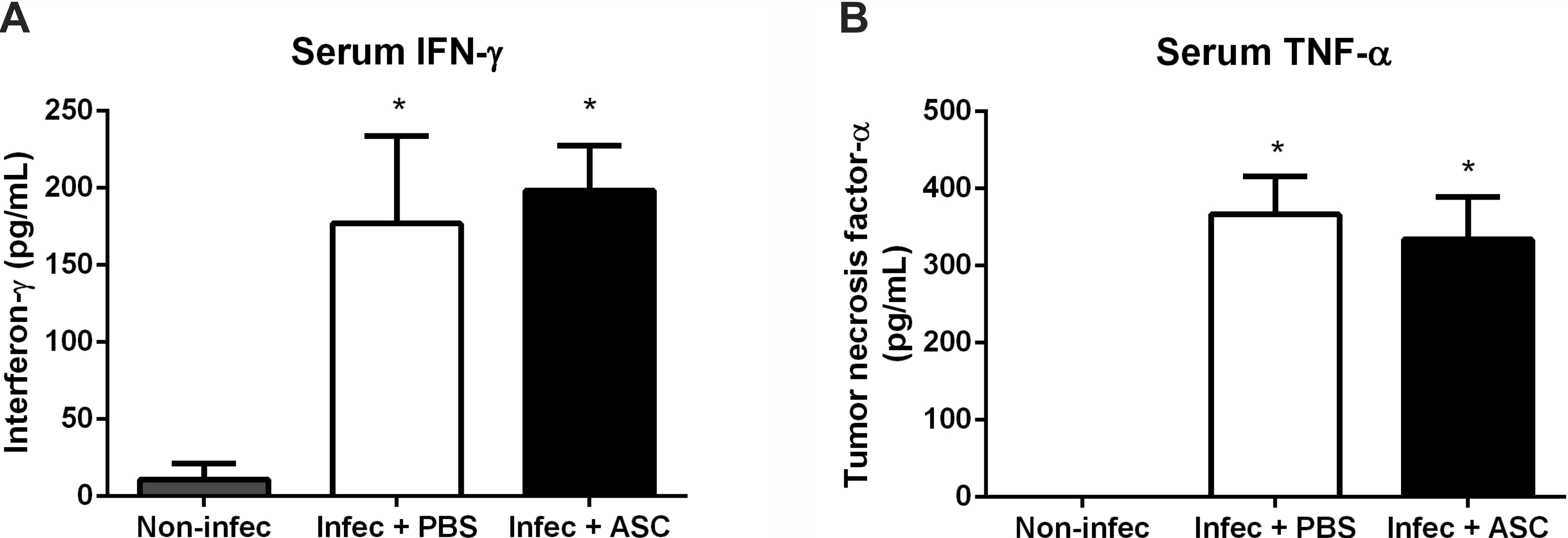

Supplement: S2 Fig — Both cytokines were increased in the infected groups when compared to non-infected animals (*p<0.05). No differences were found between placebo and ASC groups. (TIF) [file pntd.0003945.s002.tif]

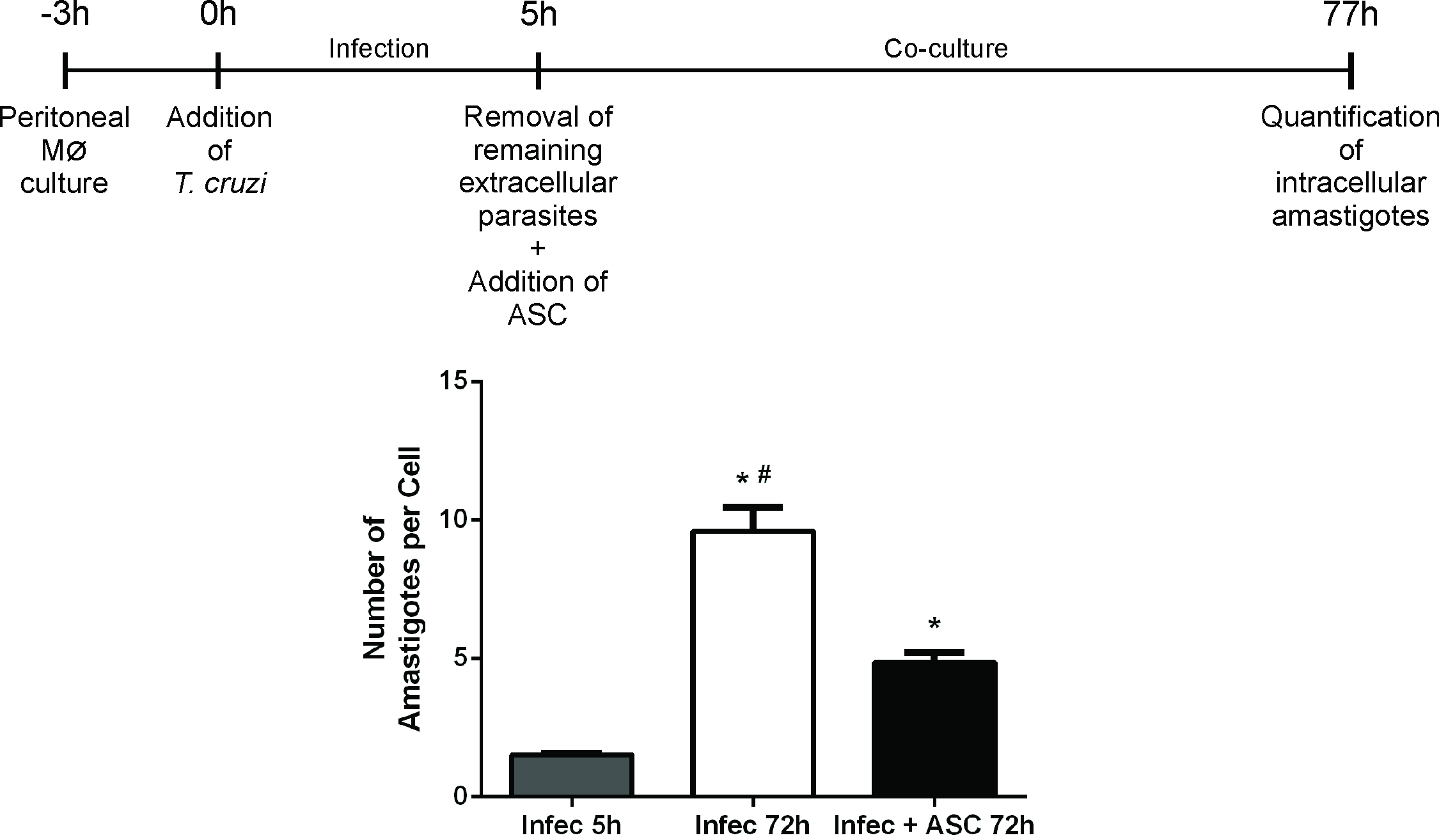

Supplement: S3 Fig — After 72 hours, there was an increase in the number of amastigotes per macrophage when compared to baseline (Infec 5h). However, co-culture with ASC reduced the number of amastigotes per cell when compared to isolated macrophages in the same time point (*p<0.05 compared to Infec 5h, #p<0.05 compared to Infec + ASC 72h). (TIF) [file pntd.0003945.s003.tif]

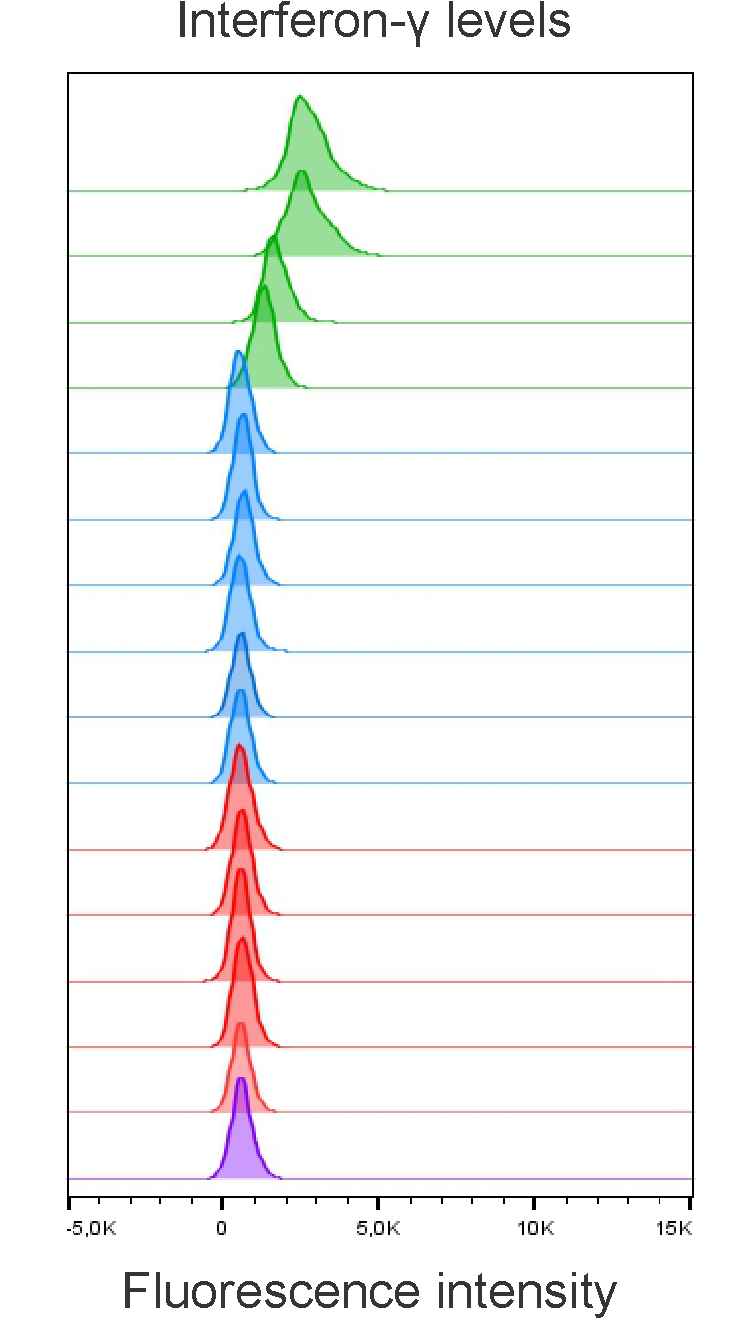

Supplement: S4 Fig — Flow cytometry histograms showing fluorescence intensity of interferon-γ in placebo (blue) and ASC-treated mice (green), as well as non-infected controls (red). The negative control is shown in purple and each histogram represents one animal. The ASC group showed a slight increase in fluorescence intensity of interferon-γ when compared to the placebo group and to non-infected mice, which were similar to the negative control. (TIF) [file pntd.0003945.s004.tif]

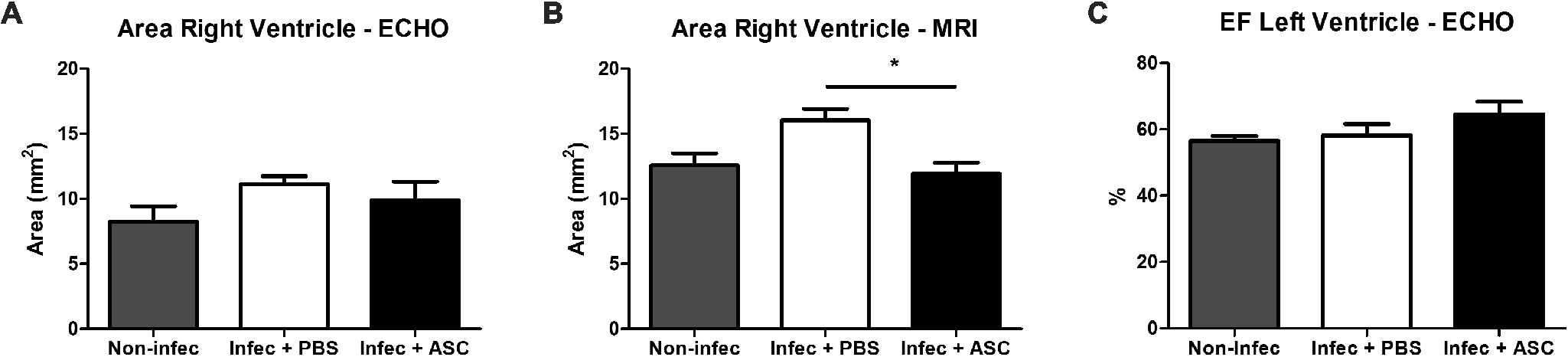

Supplement: S5 Fig — (A) No differences were found in right ventricular area when comparing non-infected, placebo-treated and ASC-treated mice. However, when the same measurement was done by MRI, there was a significant increase in RV area in placebo-treated when compared to cell-treated animals. (C) LV ejection fraction measured by echocardiography was not different among the three experimental groups. (TIF) [file pntd.0003945.s005.tif]
